# The effects of a high protein diet on indices of health and body composition – a crossover trial in resistance-trained men

**DOI:** 10.1186/s12970-016-0114-2

**Published:** 2016-01-16

**Authors:** Jose Antonio, Anya Ellerbroek, Tobin Silver, Leonel Vargas, Corey Peacock

**Affiliations:** Exercise and Sports Sciences, Nova Southeastern University, 3532 S. University Drive, University Park Plaza Suite 3532, Davie, FL 33314 USA

**Keywords:** Protein, Diet, Body composition, Nutrition, Body fat

## Abstract

**Background:**

Eight weeks of a high protein diet (>3 g/kg/day) coupled with a periodized heavy resistance training program has been shown to positively affect body composition with no deleterious effects on health. Using a randomized, crossover design, resistance-trained male subjects underwent a 16-week intervention (i.e., two 8-week periods) in which they consumed either their normal (i.e., habitual) or a higher protein diet (>3 g/kg/day). Thus, the purpose of this study was to ascertain if significantly increasing protein intake would affect clinical markers of health (i.e., lipids, kidney function, etc.) as well as performance and body composition in young males with extensive resistance training experience.

**Methods:**

Twelve healthy resistance-trained men volunteered for this study (mean ± SD: age 25.9 ± 3.7 years; height 178.0 ± 8.5 cm; years of resistance training experience 7.6 ± 3.6) with 11 subjects completing most of the assessments. In a randomized crossover trial, subjects were tested at baseline and after two 8-week treatment periods (i.e., habitual [normal] diet and high protein diet) for body composition, measures of health (i.e., blood lipids, comprehensive metabolic panel) and performance. Each subject maintained a food diary for the 16-week treatment period (i.e., 8 weeks on their normal or habitual diet and 8 weeks on a high protein diet). Each subject provided a food diary of two weekdays and one weekend day per week. In addition, subjects kept a diary of their training regimen that was used to calculate total work performed.

**Results:**

During the normal and high protein phase of the treatment period, subjects consumed 2.6 ± 0.8 and 3.3 ± 0.8 g/kg/day of dietary protein, respectively. The mean protein intake over the 4-month period was 2.9 ± 0.9 g/kg/day. The high protein group consumed significantly more calories and protein (*p* < 0.05) than the normal protein group. There were no differences in dietary intake between the groups for any other measure. Moreover, there were no significant changes in body composition or markers of health in either group. There were no side effects (i.e., blood lipids, glucose, renal, kidney function etc.) regarding high protein consumption.

**Conclusion:**

In resistance-trained young men who do not significantly alter their training regimen, consuming a high protein diet (2.6 to 3.3 g/kg/day) over a 4-month period has no effect on blood lipids or markers of renal and hepatic function. Nor were there any changes in performance or body composition. This is the first crossover trial using resistance-trained subjects in which the elevation of protein intake to over four times the recommended dietary allowance has shown no harmful effects.

## Background

There is a dearth of studies that have examined the effects of high protein diets on markers of health, body composition or performance. The International Society of Sports Nutrition’s Position Stand on Protein states that “protein intakes of 1.4–2.0 g/kg/day for physically active individuals is not only safe, but may improve the training adaptations to exercise training” [1]. Previous work from our laboratory examined a true high protein diet (4.4 g/kg/day) on measures of body composition and performance. In essence, consuming over five times the recommended daily allowance of protein had no effect on body composition in resistance-trained individuals who otherwise maintained the same training regimen. That investigation was the first interventional study to demonstrate that consuming a hypercaloric, high protein diet does not result in changes in body composition [2]. A follow-up investigation on resistance-trained men and women found that when a high protein diet is combined with a periodized heavy resistance training program, there is a subsequent loss of fat mass. Furthermore, no side effects were found via a basic metabolic panel (i.e., blood chemistry measures). Thus, the purpose of the present investigation was to determine the effects of a high protein diet (>3 g/kg/day) in resistance-trained males with extensive weight-training experience. This is the first randomized, crossover trial on high protein diets. In addition, we have performed a more extensive examination of its effects on other markers of metabolic health (i.e., blood lipids and comprehensive metabolic panel).

## Methods

### Participants

Twelve resistance-trained male subjects volunteered for this investigation. Subjects took part in a randomized crossover trial in which they consumed their habitual (i.e., normal protein) or high protein diet for two 8-week periods. Thus, there was a total treatment period of 16 weeks (i.e., 8 weeks on normal or high followed by 8 weeks on the opposite diet). Subjects came to the laboratory on three occasions: baseline, week 8 and week 16. The extra protein consumed by each subject was obtained primarily from whey protein powder. All procedures involving human subjects were approved by Nova Southeastern University’s Human Subjects Institutional Review Board in accordance with the Helsinki Declaration and written informed consent was obtained prior to participation.

### Food diary

Subjects kept a diary (i.e., three days per week for the 16 week period; two weekdays and one weekend day) of their food intake via a smartphone app (MyFitnessPal®). The use of mobile apps for dietary self-reporting has been previously used [3]. Every subject had previously used this mobile app. The MyFitnessPal® app is a database comprised of over 5 million foods that have been provided by users via entering data manually or by scanning the bar code on packaged goods. Thus, the data themselves are primarily derived from food labels (i.e., Nutrition Facts Panel) derived from the USDA National Nutrient database. Thus, in order for subjects to consume a high protein diet, protein powder (e.g., whey protein) was provided at no cost to the research subjects. However, they were not required to consume protein powder. The rest of their dietary protein was obtained from their regular food intake.

### Body composition

Height was measured using standard anthropometry and total body weight was measured using a calibrated scale. Body composition was assessed by whole body densitometry using air displacement via the Bod Pod® (COSMED USA, Concord CA). All testing was performed in accordance with the manufacturer’s instructions. Subjects were instructed to come into the lab after a 3-h fast and no exercise 24-h prior. They voided prior to testing. Subjects were tested while wearing only tight fitting clothing (swimsuit or undergarments) and an acrylic swim cap. Subjects were instructed to wear the same clothing for all testing. Thoracic gas volume was estimated for all subjects using a predictive equation integral to the Bod Pod® software. Each subject was tested at least twice per visit. The calculated value for body density used the Siri equation to estimate body composition. Data from the Bod Pod® include body weight, percent body fat, fat free mass and fat mass. All testing was done with each subject at approximately the same time of day for each of the three testing sessions. Although hydration status was not assessed, each subject was tested in an identical manner throughout the investigation. The Bod Pod was calibrated the morning of the testing session as well as between each subject.

### Performance testing

Performance testing included the one repetition maximum (1-RM) bench press and repetitions to failure (RTF) at 60 % of the bench press 1-RM. Performance tests were conducted by certified strength and conditioning specialists and followed the NSCA’s guide to tests and assessments [4]. All subjects were familiar with the performance tests prior to entering the laboratory. In general, each subject performed a movement specific warm up prior to the test (i.e., 3 sets on the bench press at progressively higher submaximal loads). Subjects then rested for 2–3 min prior to commencing the 1-RM bench press. A maximum of five attempts was attempted for the 1-RM bench press. Once the subject achieved their 1-RM, they rested for 3–5 min prior to commencing the RTF at 60 % of the 1-RM bench press. The maximal number of repetitions was subsequently determined.

### Blood analysis – comprehensive metabolic panel and blood lipids

Subjects presented after an overnight fast at a local Quest Diagnostics™ facility on three separate occasions. A blood lipid and comprehensive metabolic panel was done. This includes the following measures: glucose, blood urea nitrogen (BUN), creatinine, glomerular filtration rate, BUN/creatinine ratio, sodium, potassium, chloride, carbon dioxide, calcium, total protein, albumin, globulin, albumin/globulin ratio, total bilirubin, alkaline phosphatase, alanine transaminase, asparate transaminase, total cholesterol, high density lipoprotein cholesterol, triglycerides, low density lipoprotein cholesterol and the total cholesterol to high density lipoprotein cholesterol ratio. Quest Diagnostics performed each test according to the standard operating procedure of the company.

### Training program

Each subject followed their own strength and conditioning program. The investigators were in regular contact with each subject to ensure that each subject completed a training log. The volume load (i.e., total weight lifted per week) was determined for each 8-week period.

### Statistics

A 2-way analysis of variance (ANOVA) was used to analyze the data with a *p* <0.05 considered significant. Data are expressed as the mean ± SD. The statistical analysis was completed using Prism 6 GraphPad Software (La Jolla California).

## Results

The characteristics of the 12 male subjects in this investigation were as follows: [Mean ± SD]: age 25.9 ± 3.7 years; height 178.0 ± 8.5 cm; years of resistance training experience 7.6 ± 3.6. We did not conduct normality of data measures.

### Body composition and performance

**Fig. 1:**
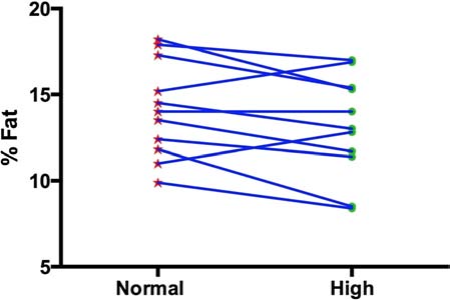
Individual changes in body fat percentage

**Fig. 2:**
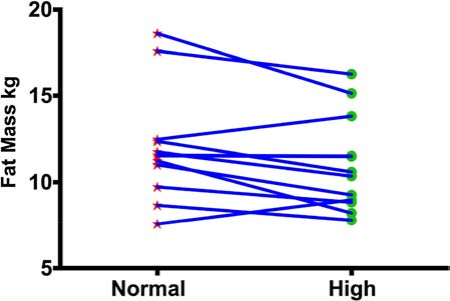
Individual changes in fat mass

**Fig. 3:**
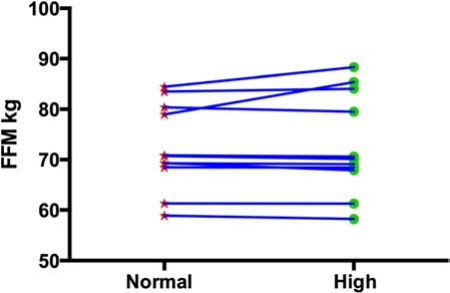
Individual changes in fat free mass

Body composition and performance data are presented in Table 1 and Figs. 1, 2 and 3 (i.e., individual changes in fat mass, FFM and % body fat). There were no significant differences between the normal and high protein groups for any of the measures.

**Table 1:** Body composition and performance

	Baseline	Normal protein	High protein
Weight kg	85.24 ± 10.83	84.43 ± 10.58	83.98 ± 10.63
Fat Mass kg	12.07 ± 3.23	12.04 ± 3.36	10.97 ± 2.89
Fat Free Mass kg	73.17 ± 9.83	72.39 ± 8.50	73.00 ± 9.93
% Body Fat	14.19 ± 3.32	14.15 ± 2.80	13.13 ± 2.98
Bench Press 1-RM kg^b^	126.4 ± 13.9	119.2 ± 17.7	122.3 ± 13.1
RTF at 60 % 1-RM BP^b^	19.9 ± 3.2	21.3 ± 5.5	21.9 ± 3.0
Volume Load kg^a^	48,783 ± 19,506	50,578 ± 18,881	48,989 ± 15,388

Data are mean ± SD. *n* = 11 (one subject’s body composition data was incomplete)

*BP* bench press, *kg* kilograms, *RTF* repetitions to failure

^a^Volume Load is calculated as the total amount of weight lifted per week (i.e., repetitions x weight for each set). ^b^ *N* = 7 (four subjects could not do the exercise tests due to overuse injuries)

### Diet

**Fig. 4:**
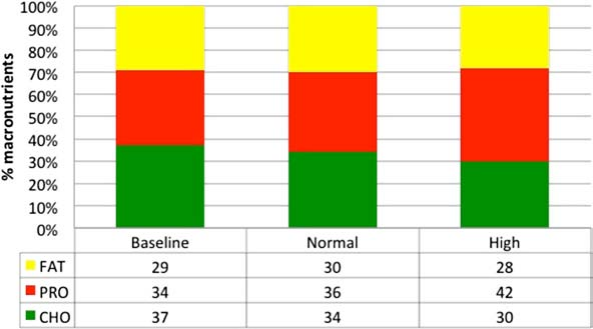
The Macronutrient percentages of each group

There were significant differences in total energy and protein intake between the high protein, normal protein group and baseline (high > normal and baseline; *p* < 0.05) (Table 2). The mean percentage of macronutrients (CHO:PRO:Fat) were as follows: Baseline - 37:34:29, Normal - 34:36:30 and High - 30:42:28 (Fig. 4).

**Table 2:** Dietary intake

	Baseline	Normal protein	High protein
Kcal	2453 ± 352	2534 ± 343	2903 ± 415^*#^
CHO g	226 ± 81	220 ± 65	219 ± 78
PRO g	190 ± 76	212 ± 65	271 ± 61^*#^
Fat g	80 ± 27	86 ± 28	88 ± 16
Kcal/kg/day	30.4 ± 7.3	31.6 ± 7.5	35.0 ± 4.6^*^
CHO g/kg/day	2.7 ± 1.0	2.6 ± 1.0	2.7 ± 1.0
PRO g/kg/day	2.3 ± 1.0	2.6 ± 0.8	3.3 ± 0.8^*#^
Fat g/kg/day	1.0 ± 0.4	1.1 ± 0.4	1.0 ± 0.2
Cholesterol mg/day	542 ± 359	464 ± 285	780 ± 566
Sodium mg/day	2892 ± 1125	3175 ± 971	3484 ± 766
Sugars g/day	49 ± 33	50 ± 27	63 ± 21
Fiber g/day	27 ± 16	27 ± 18	30 ± 12

Data are mean ± SD. *n* = 12

*CHO* carbohydrate, *PRO* protein, *g* grams, *kg* kilograms, *d* days, *HP* high protein, *NP* normal protein

^*^ *P* < 0.05 – denotes significantly different than baseline. ^#^ *P* < 0.05 – denotes significantly different than normal protein

### Blood analysis

There were no changes in any of the variables regarding blood lipids and a comprehensive metabolic panel (Tables 3 and 4). We examined the two individuals with the highest recorded protein intakes (4.66 and 6.59 g/kg/day) and found no deleterious effects on renal function in either individual (Table 5).

**Table 3:** Comprehensive metabolic panel

	Baseline	Normal protein	High protein	Reference range
Glucose mg/dL	83 ± 12	85 ± 14	84 ± 19	65–99
BUN mg/dL	22 ± 5	23 ± 5	23 ± 6	7–25
Creatinine mg/dL	1.1 ± 0.2	1.1 ± 0.1	1.1 ± 0.2	0.60–1.35
eGFR ml/min/1.73 m2	96 ± 20	102 ± 18	101 ± 18	§
BUN/Creatinine ratio	19.4 ± 5.4	21.2 ± 4.5	20.5 ± 2.8	6–22
Sodium mmol/L	139 ± 2	138 ± 2	138 ± 1	135–146
Potassium mmol/L	4.3 ± 0.4	4.2 ± 0.3	4.3 ± 0.2	3.5–5.3
Chloride mmol/L	103 ± 2	102 ± 1	102 ± 3	98–110
Carbon Dioxide mmol/L	27 ± 2	27 ± 4	27 ± 2	19–30
Calcium mg/dl	9.7 ± 0.2	9.6 ± 0.3	9.6 ± 0.3	8.6–10.3
Total Protein g/dL	7.2 ± 0.4	7.2 ± 0.3	7.1 ± 0.4	6.1–8.1
Albumin g/dL	4.7 ± 0.2	4.6 ± 0.2	4.6 ± 0.3	3.6–5.1
Globulin g/dL	2.5 ± 0.3	2.6 ± 0.3	2.6 ± 0.3	1.9–3.7
Albumin/Globulin ratio	1.9 ± 0.2	1.8 ± 0.2	1.8 ± 0.2	1.0–2.5
Total Bilirubin mg/dL	0.7 ± 0.3	0.7 ± 0.2	0.7 ± 0.3	0.2–1.2
Alkaline Phosphatase U/L	65 ± 17	66 ± 20	65 ± 16	40–115
AST U/L	28 ± 9	27 ± 6	27 ± 6	10–40
ALT U/L	29 ± 19	27 ± 9	28 ± 10	9–46

Data are mean ± SD. *n* = 12. *ALT* alanine transaminase, *AST* aspartate transaminase, *BUN* blood urea nitrogen, *eGFR* estimated glomerular filtration rate (§ normal values: ≥60 ml/min/1.73 m^2^). There were no differences between any of the groups

**Table 4:** Lipid panel

	Baseline	Normal protein	High protein	Reference range
Total Cholesterol mg/dL	161 ± 30	143 ± 24	152 ± 31	125–200
HDL Cholesterol mg/dL	48 ± 16	46 ± 20	48 ± 11	≥40
Triglycerides mg/dL	64 ± 18	57 ± 25	64 ± 28	<150
LDL Cholesterol mg/dL	100 ± 36	86 ± 26	91 ± 26	<130
CHOL/HDL-C ratio	4.1 ± 3.2	4.4 ± 4.5	3.2 ± 0.7	≤5.0

Data are mean ± SD. *n* = 12. There were no differences between any of the groups

**Table 5:** Case reports - renal function on two subjects with the highest protein intakes

	Baseline	Normal protein	High protein	Reference range
Subject 1				
PRO intake g/kg/d	3.98	4.18	6.59	724 % > than the RDA
BUN mg/dL	25	34	14	7–25
Creatinine mg/dL	1.26	1.09	0.96	0.60–1.35
eGFR ml/min/1.73 m2	88	105	122	§
BUN/Creatinine ratio	19.8	31.2	14.6	6–22
Subject 2				
PRO intake g/kg/d	2.56	3.61	4.66	483 % > than the RDA
BUN mg/dL	22	26	20	7–25
Creatinine mg/dL	0.97	0.97	1.02	0.60–1.35
eGFR ml/min/1.73 m2	125	126	119	§
BUN/Creatinine ratio	22.7	26.8	19.6	6–22

Data are mean ± SD. *BUN* blood urea nitrogen, *eGFR* estimated glomerular filtration rate (§ normal values: ≥60 ml/min/1.73 m^2^), *PRO* protein, *RDA* recommended dietary allowance

## Discussion

This is the third investigation from our laboratory that has examined the effects of a high protein diet (i.e., > 2 g/kg/day). Previously published work has shown that consuming a high protein diet (4.4 g/kg/day) does not significantly affect body composition (i.e., no statistically significant change in FFM, fat mass or % body fat) in trained individuals who do not substantially change their exercise regimen [2]. On the other hand, a follow-up study found that a high protein diet (3.4 g/kg/d) in conjunction with a periodized heavy resistance training program can favorably alter body composition [5]. It should be noted that although the previous investigation used resistance-trained subjects, training experience varied greatly. The current study used only highly experienced resistance-trained males (i.e., ~8 years of training experience with a mean 1-RM bench press of 126 kg). They could lift on average ~1.5 times their body weight. The subjects in the current study also had more than twice the resistance training experience as those in our prior investigation [5].

Similar to our first investigation [2], the current study found no statistically significant effects of a high protein diet on body composition, 1-RM bench press strength or muscular endurance (RTF at 60 % of the 1-RM bench press) when compared to the normal protein group. Although our subjects consumed approximately 400 additional calories daily for eight weeks, there were no significant changes in fat mass despite the fact that there were no changes in their exercise regimen.

With highly trained subjects, it is important that one examine individual data points. Nine of 11 subjects demonstrated a decrease in fat mass during the high protein diet phase. Two subjects showed an increase in fat mass. Relying on mean changes, particularly for trained subjects, is not an ideal way to understand the adaptive response to diet and/or exercise. Both mean and individual data points provide a much clearer picture of how high protein intakes affect various measures. Certainly, the small sample size (i.e., the study was underpowered) is likely the reason for the lack of statistical significance regarding fat mass. Nevertheless, the intriguing finding in the current study is that overfeeding on protein does not typically have an adverse effect on body composition.

The possible explanations for the lack of weight gain in our subjects include the following: changes in the thermic effect of exercise (TEE) as well as non-exercise activity thermogenesis (NEAT) might account in part for the slight decrease in percent body fat in the high protein diet group [6, 7]. Ostensibly, NEAT can vary by as much as 2000 cal between individuals [7]. Thus, it is possible that in our group of well-trained subjects, NEAT could account for some of the additional energy expenditure. In addition to NEAT, dietary protein itself has profound thermic effect. Protein’s thermic effect of feeding (TEF) is 19–23 % in both obese and lean individuals whereas carbohydrate is approximately 12–14 % [8]. A high protein diet (45 % total kcal) elicits a 30 % greater TEF than an isocaloric low protein (15 % total kcal) in active females [9]. The subjects in our study did not alter fat or carbohydrate intake; thus, that could not be an explanation for changes in body composition. Thus, one might speculate that the high protein diet group experienced a combination of enhanced TEF, TEE and NEAT. Furthermore, recent animal data suggest that a high-protein diet might reduce fat mass by inhibiting lipogenesis in the liver [10].

In conjunction with our prior work, we further examined blood lipids as well as other markers of health. We found no deleterious effects of high protein consumption. There were no changes in blood lipids as well as renal or hepatic function. On average, subjects in this investigation consumed ~3 g of protein per kilogram of body weight daily for four months. In fact, the subjects with the two highest levels of protein intake showed no changes in renal function despite exceeding the RDA by 483–724 %. Thus, it is evident that even at very high protein intakes, there are no harmful side effects.

It is worth noting that the fiber intake of our subjects was 27–30 g per day. The average fiber intake in the United States is 16 g per day [11]. Therefore, the notion that a high protein diet and adequate fiber consumption is mutually exclusive is not supported by our data. One might speculate the combination of higher protein and fiber intake might assist in promoting fat loss [12]. Thus, the fact that our subjects were healthy (i.e., blood lipids, renal and hepatic function, etc.) may have been due partially to their fiber intake. It is known that higher fiber intakes are associated with a lower risk of cardiovascular disease [13]. Furthermore, the cholesterol intake of our subjects were as much as 160 % greater than the typical recommendation of 300 mg per day [14]. Thus it is apparent that in this select sample of highly trained males, cholesterol intake has little effect on blood measures of cardiovascular health.

The strengths of our investigation included the use of highly trained subjects and the fact that we used a crossover design. Thus, each subject could be compared to himself. The small sample size as well as the lack of control for the training program are certainly confounding variables. Also, we did not ascertain the hydration status of each subject. This could indeed affect our body composition assessment [15]. It is known that there may be a decrease in FFM if the subject went from a hydrated to a dehydrated state. To insure that this was minimized, we did follow identical pre- and post-testing body composition procedures.

## Conclusion

In conclusion, this is the first randomized crossover trial in resistance-trained subjects that examined the effects of consuming a high protein diet over the course of several months on markers of performance, health and body composition. This study as well as previous work from our lab suggests that gains in body fat are unlikely to occur with protein overfeeding in conjunction with an increase in total energy intake [2].

Limitations of this study include the use of dietary self-reports. It has been posited that underreporting of dietary intake is a severe confounding variable in studies that involve a dietary intervention. Interestingly, this is not a universal finding. American women consistently underreport their caloric intake in contrast with only 10 % of Egyptian women [16]. Novotny et al. found that that a sex difference existed in underreporting (i.e., women underreport more than men) [17]. Much of the additional protein consumed by the subjects in the current study was in the form of protein powder; therefore it would seem reasonable to assume that our male subjects could provide an accurate dietary recall with such a simple dietary addition. Furthermore, from an entirely pragmatic perspective, the use of dietary recalls is the best option to assess food intake in free-living individuals. Future work should examine very highly trained athletes who undergo cycles of varying protein intake over a period of several months or years. This would at least provide information in terms of whether the highly trained respond more so to a change in training stimulus, diet or a combination of both.
